# A practical risk stratification system based on ultrasonography and clinical characteristics for predicting the malignancy of soft tissue masses

**DOI:** 10.1186/s13244-024-01802-9

**Published:** 2024-09-19

**Authors:** Ying-Lun Zhang, Meng-Jie Wu, Yu Hu, Xiao-Jing Peng, Qian Ma, Cui-Lian Mao, Ye Dong, Zong-Kai Wei, Ying-Qian Gao, Qi-Yu Yao, Jing Yao, Xin-Hua Ye, Ju-Ming Li, Ao Li

**Affiliations:** 1 Department of Ultrasound, The First Affiliated Hospital of Nanjing Medical University, Nanjing, China; 2 Department of Ultrasound, The Affiliated Drum Tower Hospital of Nanjing University Medical School, Nanjing, China; 3 Department of Orthopedics, The First Affiliated Hospital of Nanjing Medical University, Nanjing, China; 4 Present Address: Department of Ultrasound, The Affiliated Drum Tower Hospital of Nanjing University Medical School, Nanjing, China

**Keywords:** Diagnosis, Soft tissue mass, Ultrasonography

## Abstract

**Objective:**

To establish a practical risk stratification system (RSS) based on ultrasonography (US) and clinical characteristics for predicting soft tissue masses (STMs) malignancy.

**Methods:**

This retrospective multicenter study included patients with STMs who underwent US and pathological examinations between April 2018 and April 2023. Chi-square tests and multivariable logistic regression analyses were performed to assess the association of US and clinical characteristics with the malignancy of STMs in the training set. The RSS was constructed based on the scores of risk factors and validated externally.

**Results:**

The training and validation sets included 1027 STMs (mean age, 50.90 ± 16.64, 442 benign and 585 malignant) and 120 STMs (mean age, 51.93 ± 17.90, 69 benign and 51 malignant), respectively. The RSS was constructed based on three clinical characteristics (age, duration, and history of malignancy) and six US characteristics (size, shape, margin, echogenicity, bone invasion, and vascularity). STMs were assigned to six categories in the RSS, including no abnormal findings, benign, probably benign (fitted probabilities [FP] for malignancy: 0.001–0.008), low suspicion (FP: 0.008–0.365), moderate suspicion (FP: 0.189–0.911), and high suspicion (FP: 0.798–0.999) for malignancy. The RSS displayed good diagnostic performance in the training and validation sets with area under the receiver operating characteristic curve (AUC) values of 0.883 and 0.849, respectively.

**Conclusion:**

The practical RSS based on US and clinical characteristics could be useful for predicting STM malignancy, thereby providing the benefit of timely treatment strategy management to STM patients.

**Critical relevance statement:**

With the help of the RSS, better communication between radiologists and clinicians can be realized, thus facilitating tumor management.

**Key Points:**

- There is no recognized grading system for STM management.
- A stratification system based on US and clinical features was built.
- The system realized great communication between radiologists and clinicians in tumor management.

**Graphical Abstract:**

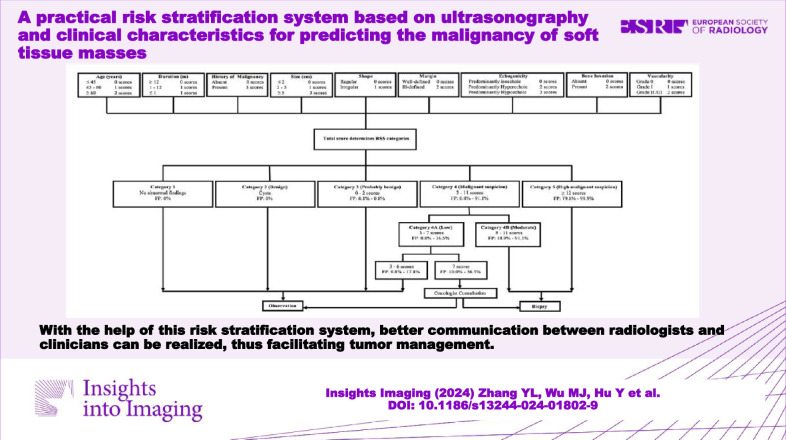

## Introduction

Soft tissue masses (STMs), a kind of common clinical disease with a morbidity of approximately 300 cases/100,000 people, can occur in all body parts [1]. Among them, benign STMs far outnumber malignant STMs, with malignant cases accounting for less than 1% [2]. Sadly, a malignant STM has a poor prognosis, increasing the patient’s emotional and financial burdens [3]. Therefore, avoiding over-examination and intervention of benign cases and focusing on malignant cases are crucial for STM management. MRI is the primary modality for STM diagnosis, but its time consumption, cost, and inaccessibility for patients with claustrophobia or metal stents greatly limit its clinical use [4]. As a first-line alternative, ultrasonography (US) is convenient and valuable in diagnosing STMs [5–7].

Currently, the US diagnosis of STMs has been divided into ultrasomics and multimodal US. Although ultrasomics can transform images into high-throughput, extractable features that allow an objective analysis of STMs, the data processing is complex and unsuitable for promotion [8]. Multimodal US combines gray-scale US with novel techniques such as elastography and contrast-enhanced US and has an accuracy range of 77–88% [5, 6, 9–11]. However, its application is limited due to the differences in equipment and observers’ experience. Worse still, no consensus exists on the suspicious image features for malignancy. Therefore, standardization of US image acquisition and interpretation of STMs is necessary for widespread application. The broadly used breast imaging reporting and data system (BI-RADS) and thyroid imaging reporting and data system (TI-RADS) that define malignant features and classify the degree of malignancy can help manage breast or thyroid lesions and can serve as references for establishing a risk stratification system (RSS) for STMs [12, 13]. To our knowledge, no US features-based RSS currently exists for STM management. Due to disease heterogeneity, adopting the TI-RADS or BI-RADS without modification is inappropriate. Specific evaluation and scoring of risk US features for STMs is still needed. Moreover, clinical features such as age and growth speed have been reported to be important in the diagnosis of STMs [14]. The integration of clinical features with US features may contribute to the reliability of the system, as well as clinical utility.

This study aimed to establish a practical RSS for predicting the malignancy of STMs using retrospective data of US and clinical information and validated the system on independent multicenter datasets. This strategy has the potential to achieve precise preoperative prediction of STM malignancy and help guide treatment planning.

## Methods

### Patients

This retrospective study was approved by the ethics committee of Hospital #1 (The First Affiliated Hospital of Nanjing Medical University, Nanjing, China) with an exemption for written informed consent. Informed consent for biopsy or surgery was obtained from all the patients. The Declaration of Helsinki was followed in this study.

From April 2018 to April 2023, patients with suspicious STMs were reviewed in Hospital #1. Patients were included in this study if they (1) underwent US examination with satisfactory image quality and (2) had a definitive histopathologic result that showed whether the tumor was benign, intermediate, or malignant. Exclusion criteria were that (1) patients had a history of biopsy or treatment of masses before US examination; (2) the intervals between US examination and biopsy or surgery exceeded two weeks; (3) clear records of clinical information were lacking; and (4) masses were located in the thyroid, breast, salivary glands, lymph nodes, gynecological system, and retroperitoneal system. Patients with typical cysts were also excluded because we directly defined Category 1 as having no abnormal findings and Category 2 as benign, showing typical cysts based on BI-RADS and TI-RADS.

**Fig. 1:**
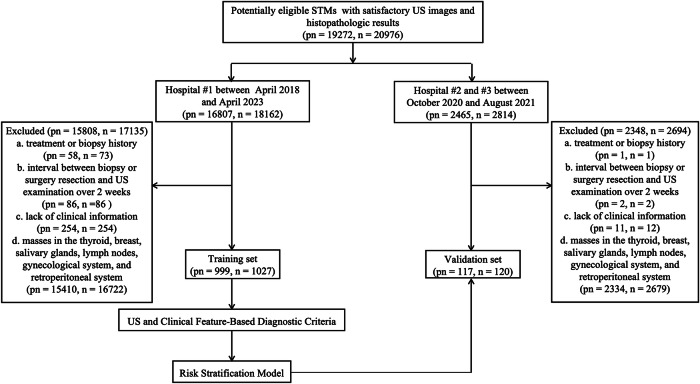
Flowchart of the eligible patients and number of STMs. Note: typical cysts are directly defined as Category 2 in this study so they are not included in the flowchart. n, number of STMs; pn, number of patients; STMs, soft tissue masses; US, ultrasonography

Ultimately, 1027 STMs in 999 patients (mean age = 50.90 ± 16.64 years, range 4–92 years, male/female ratio 1:1.08) formed the training set. The validation set included 120 STMs in 117 patients (mean age = 51.93 ± 17.90 years, range 9–84 years, male/female ratio 1:1.14) enrolled in Hospital #2 (The Affiliated Drum Tower Hospital of Nanjing University Medical School, Nanjing, China) and Hospital #3 (Shanghai Tenth People’s Hospital of Tongji University, Shanghai, China) from October 2020 to August 2021. The inclusion and exclusion criteria were the same as the training set. The participant selection flowchart is shown in Fig. 1.

### Clinical information collection

All clinical information was obtained from the medical records database. The collected clinical features were determined based on previous literature and the clinical experience of oncologists in Hospital #1 [14]. The following features were collected: sex: male or female; age (years): ≤ 45, 45–60, or ≥ 60; pain: absent or present; duration: months since the mass was discovered, including ≤ 1, 1–12, or ≥ 12; history of malignancy: absent or present; location: head and neck, trunk and hip, or limbs.

### Imaging acquisition and interpretation

US examinations were performed using various US instruments, such as LOGIQ E9 (GE Healthcare, Pittsburgh, Pa, USA), Acuson S3000 (Siemens Healthineers, Erlangen, Bavaria, Germany), or EPIQ7 (Philips Medical Systems, Bothell, WA, USA) with linear or convex transducers. The specific scanning protocol criteria were transverse and longitudinal scanning of each STM with full mass exposure. Trapezoidal or panoramic imaging was used when necessary. The maximum transverse- and longitudinal-section grayscale US images of each STM were routinely recorded. Furthermore, the best color Doppler flow imaging (CDFI) images on the same transverse sections were recorded. All examinations were performed by radiologists with over two years of experience in the musculoskeletal US from three separate hospitals, and two radiologists (Y.-L.Z. and M.-J.W.) in Hospital #1 with 2–5 years of experience, who were blind to the clinical information and mass pathology reviewed all grayscale US and CDFI images from all the hospitals. Disagreements were resolved through discussion under the supervision of a senior radiologist (A.L.) with over five years of experience in Hospital #1. Criteria were made based on previous literature and radiologists’ experiences [8, 15–20]. The following features were recorded: layer (relative to the investing fascia): superficial or deep; size: ≤ 2 cm, 2–5 cm, or ≥ 5 cm; shape: regular (ovoid to round) or irregular (not ovoid to round); margin: well-defined (smooth and clear) or ill-defined (jagged, spiculated, blurred, slightly lobulated, or extra-mass extended); echogenicity of the solid and noncalcified component of a mass (relative to adjacent muscle): predominantly isoechoic, predominantly hyperechoic, or predominantly hypoechoic; composition: predominantly solid (cystic portion ≤ 10%), mixed (cystic portion > 10% but ≤ 50%), or predominantly cystic (cystic portion > 50%); calcification: absent or present; bone invasion: absent or present; vascularity: grade 0 (no blood flow in the mass), grade I (only a tiny amount of blood flows with 1–2 punctuate or rod-shaped blood flows), grade II (moderate blood flows with 3–4 punctuate blood flows or a vital blood vessel), or grade III (rich blood flows with more than one important blood vessels).

### Reference standard

Histopathologic results of biopsy or surgery served as the reference standards. Malignant STMs included malignant and intermediate STMs defined by the 2020 World Health Organization (WHO) classification of soft tissue tumors [1], metastatic tumors, and hematologic malignancies. Meanwhile, benign STMs referred to benign STMs defined by the 2020 WHO classification.

### RSS construction and statistical analysis

This study concentrated on creating the RSS Category 3–5 and included six main steps of statistical analysis. (1) All clinical and US features were categorized based on the predetermined criteria. The differences in malignancy frequency and clinical and US features between the training and validation sets were determined via Chi-square tests. (2) The clinical and US features in the training set were assessed by Chi-square tests. (3) The significant features acquired from the univariate analysis were included in the multivariable analysis. The binary logistic regression model determined the risk factors, and their β coefficients and 95% confidence interval (CI) were calculated. A regression equation for fitted probabilities (FP) was gained as well [21]. Ten-fold cross-validation was used to evaluate the multivariable logistic regression model [22]. (4) β coefficients for risk factors from the logistic regression model were used for risk score analyses. The β coefficients were standardized to make the value of the smallest one equal 1. Risk scores for each risk factor were assessed as the closest integer of the standardized β values [23]. An individual’s risk score was then determined by summing the scores of each risk factor. (5) RSS was built based on the risk scores and the FP, and the actual malignancy rates were also calculated. The linear relationships between both the malignancy probabilities and risk scores, as well as the malignancy probabilities and the RSS categories were assessed by Cochran–Armitage trend tests. (6) Finally, the area under the curves (AUCs) with 95% CI, accuracy, sensitivity, and specificity of the RSS in the two sets were calculated.

The R software (version 4.3.1, R Project for Statistical Computing, www.r-project.org) was used for 10-fold cross-validation and the Cochran–Armitage trend tests. Chi-square tests and logistic regression were performed using SPSS 26.0 software (IBM, Ehningen, Germany). Inter-observer agreements of US features in the two sets were assessed by Kappa statistics. *p* < 0.05 was indicative of a statistically significant difference, and all reported *p* values were two-sided.

## Results

### Clinical and US features of patients

Overall, the malignancy frequency distribution, clinical features, and the majority of US features were similar in the two sets, with a difference in vascularity (*p* = 0.044) (Table 1). The inter-observer agreements of US features in the two sets are shown in Supplementary Table 1. In the training set, there were 442 malignant (233 confirmed surgically and 209 confirmed by biopsies) and 585 benign (469 confirmed surgically and 116 confirmed by biopsies) STMs. In the validation set, there were 51 malignant (21 confirmed surgically and 30 confirmed by biopsies) and 69 benign (32 confirmed surgically and 37 confirmed by biopsies) STMs. The prevalent benign STMs were lipoma (*n* = 200 vs *n* = 23, the training set vs the validation set), schwannoma (*n* = 93 vs *n* = 12, the training set vs the validation set), and hemangioma (*n* = 72 vs *n* = 12, the training set vs the validation set). The malignant STMs in the training set contained 269 malignant soft tissue tumors, with common types being aggressive fibromatosis (*n* = 35), myxofibrosarcoma (*n* = 30), and atypical lipomatous tumor (*n* = 26), 125 metastatic tumors, and 48 hematologic malignancies. In comparison, the malignant STMs in the validation sets contained 31 malignant soft tissue tumors, with common types being myxofibrosarcoma (*n* = 10), aggressive fibromatosis (*n* = 4), and atypical lipomatous tumor (*n* = 4), 18 metastatic tumors, and 2 hematologic malignancies.

**Table 1:** Malignancy frequency distributions, clinical and US features of patients in the training and external validation sets

Features	Training set, (*n* = 1027)	Validation set, (*n* = 120)	*p* value
Malignancy frequency	442	51	0.910
Sex			0.781
Male	493	56	
Female	534	64	
Age (year)			0.592
≤ 45	337	36	
45–60	344	38	
≥ 60	346	46	
Pain			0.731
Absent	709	81	
Present	318	39	
Duration (month)			0.070
≥ 12	290	46	
1–12	430	44	
≤ 1	307	30	
History of malignancy			0.863
Absent	803	93	
Present	224	27	
Location			0.741
Head or neck	146	14	
Trunk or hip	360	44	
Limbs	521	62	
Layer			0.247
Superficial	336	33	
Deep	691	87	
Size (cm)			0.649
≤ 2	189	24	
2–5	425	53	
≥ 5	413	43	
Shape			0.895
Regular	597	69	
Irregular	430	51	
Margin			0.837
Well-defined	675	80	
Ill-Defined	352	40	
Echogenicity			0.090
Predominantly isoechoic	104	5	
Predominantly hyperechoic	141	20	
Predominantly hypoechoic	782	95	
Composition			0.236
Predominantly cystic	6	2	
Mixed	79	6	
Predominantly solid	942	112	
Calcification			0.896
Absent	964	113	
Present	63	7	
Bone invasion			0.708
Absent	967	114	
Present	60	6	
Vascularity			0.044*
Grade 0	338	40	
Grade I	168	30	
Grade II	228	27	
Grade III	293	23	

*n* number, *US* ultrasonography

* Indicates a significant difference between the two sets

### Construction of RSS

The univariate analysis showed a significant correlation between tumor malignancy and sex, age, pain, duration, history of malignancy, layer, size, shape, margin, echogenicity, calcification, bone invasion, and vascularity (Table 2). The binary logistic regression analysis was performed to assess independent risk factors of malignancy, which included age (45–60 or ≥ 60 years old), duration (1–12 months or ≤ 1 month), history of malignancy, size (2–5 or ≥ 5 cm), irregular shape, ill-defined margin, echogenicity (predominantly hyperechoic or hypoechoic), bone invasion, and vascularity (grade I or II or III) (Table 2). The logistic regression model’s AUC, accuracy, sensitivity, and specificity were 0.917 (95% CI: 0.900–0.933), 0.835, 0.873, and 0.798, respectively. The Hosmer–Lemeshow test indicated the absence of statistical significance (X^2^ = 6.325, *p* = 0.611). After 10-fold cross-validation, the model displayed an excellent predictive performance with a mean AUC, accuracy, sensitivity, and specificity of 0.902 (95% CI: 0.883–0.920), 0.815, 0.854, and 0.702, respectively.

**Table 2:** Association between STM malignancy and various clinical and US features

Features	Benign, (*n* = 585)	Malignant, (*n* = 442)	*p* value^*^	β (95% CI)^†^	*p* value^†^	Score^‡^
Sex			0.034			
Male	264	229		NA	NA	NA
Female	321	213		NA	NA	NA
Age (year)			< 0.001			
≤ 45	253	84		NA	NA	0
45–60	205	139		0.728 (0.271–1.194)	0.002	1
≥ 60	127	219		1.534 (1.072–2.011)	< 0.001	2
Pain			< 0.001			
Absent	432	277		NA	NA	NA
Present	153	165		NA	NA	NA
Duration (month)			< 0.001			
≥ 12	229	61		NA	NA	0
1–12	207	223		0.890 (0.429–1.361)	< 0.001	1
≤ 1	149	158		0.992 (0.502–1.491)	< 0.001	1
History of malignancy			< 0.001			
Absent	523	280		NA	NA	0
Present	62	162		1.842 (1.368–2.336)	< 0.001	3
Location			0.684			
Head or neck	86	60		NA	NA	NA
Trunk or hip	209	151		NA	NA	NA
Limbs	290	231		NA	NA	NA
Layer			< 0.001			
Superficial	271	65		NA	NA	NA
Deep	314	377		NA	NA	NA
Size (cm)			< 0.001			
≤ 2	160	29		NA	NA	0
2–5	267	158		0.966 (0.402–1.558)	0.001	1
≥ 5	158	255		2.119 (1.526–2.744)	< 0.001	3
Shape			< 0.001			
Regular	449	148		NA	NA	0
Irregular	136	294		0.709 (0.320–1.098)	< 0.001	1
Margin			< 0.001			
Well-defined	505	170		NA	NA	0
Ill-Defined	80	272		1.729 (1.326–2.144)	< 0.001	2
Echogenicity			< 0.001			
Predominantly isoechoic	95	9		NA	NA	0
Predominantly hyperechoic	109	32		1.195 (0.273–2.205)	0.015	2
Predominantly hypoechoic	381	401		1.555 (0.714–2.497)	0.001	2
Composition			0.263			
Predominantly cystic	5	1		NA	NA	NA
Mixed	49	30		NA	NA	NA
Predominantly solid	531	411		NA	NA	NA
Calcification			0.009			
Absent	559	405		NA	NA	NA
Present	26	37		NA	NA	NA
Bone invasion			< 0.001			
Absent	581	386		NA	NA	0
Present	4	56		1.363 (0.266–2.698)	0.025	2
Vascularity			< 0.001			
Grade 0	284	54		NA	NA	0
Grade I	101	67		0.978 (0.423–1.538)	< 0.001	1
Grade II	105	123		1.256 (0.739–1.783)	< 0.001	2
Grade III	95	198		1.606 (1.082–2.143)	< 0.001	2

*CI* confidence interval, *NA* not applicable, *n* number, *STMs* soft tissue masses, *US* ultrasonography

* Determined with Chi-square tests

^†^ Determined with logistic regression analysis

^‡^ Scoring criteria for significant risk factors were based on the rounded standardized β coefficients. As the lowest β value was 0.709, its multiplication by 1.41 made it close to 1. To be standardized, all other β values were multiplied by 1.41 and were rounded to the closest integer

**Fig. 2:**
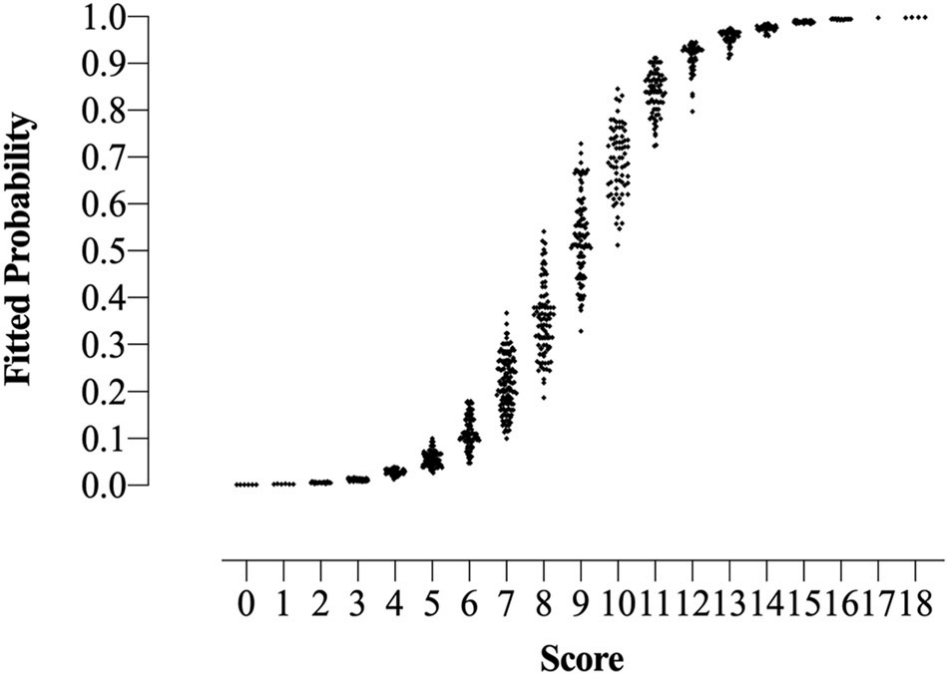
The scatter plot of FP by total scores. Note: the FP tended to increase as total scores increased

In the risk score analysis, the scores for risk factors are shown in Table 2 as well. Table 3 and Fig. 2 show the FP for the total scores. The Cochran–Armitage trend test revealed that the FP increased when the scores increased (*Z* = 22.350, *p* < 0.001). Under the cut-off value of 7.5, the risk score model indicated an AUC of 0.912 (95% CI: 0.895–0.930), an accuracy of 0.832, a sensitivity of 0.910, and a specificity of 0.762.

**Table 3:** Malignant STMs numbers, total STMs numbers, the corresponding FP, and actual malignancy rates by total scores

Score	Malignancy, *n*	Total, *n*	FP	Malignancy rates
0	0	6	0.001	0.000
1	0	6	0.002–0.003	0.000
2	0	22	0.004–0.008	0.000
3	2	59	0.008–0.017	0.034
4	3	71	0.013–0.039	0.042
5	5	94	0.027–0.099	0.053
6	8	91	0.048–0.178	0.088
7	22	137	0.100–0.365	0.161
8	41	88	0.189–0.539	0.466
9	52	101	0.326–0.805	0.515
10	56	72	0.512–0.845	0.778
11	58	75	0.726–0.911	0.773
12	50	58	0.798–0.946	0.862
13	56	58	0.912–0.976	0.966
14	38	38	0.959–0.985	1.000
15	32	32	0.985–0.992	1.000
16	14	14	0.993–0.996	1.000
17	1	1	0.997	1.000
18	4	4	0.998–0.999	1.000

*FP* fitted probabilities, *n* number of STMs, *STMs* soft tissue masses

**Fig. 3:**
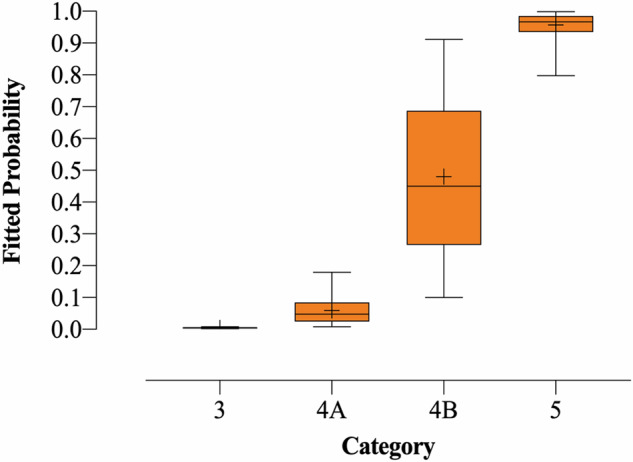
The box plot of FP by detailed categories. Note: the upper edge of the box is the 75th percentile of the FP, and the lower edge represents the 25th percentile. The line in the box represents the medians, and points indicated by + in the box represent the means. The lower and upper ends of vertical lines represent the minimum and maximum values of the FP

**Fig. 4:**
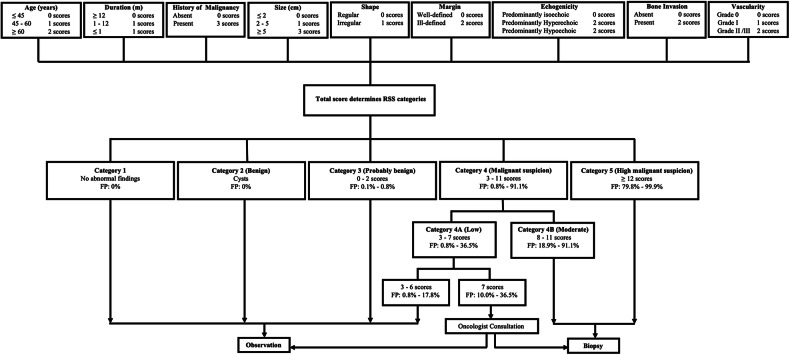
Workflow shows the detailed categories of RSS and management recommendations. FP, fitted probabilities; RSS, risk stratification system

With these findings, we created RSS Category 3 (score: 0–2, FP: 0.001–0.008, actual malignancy rate: 0), 4A (score: 3–7, FP: 0.008–0.365, actual malignancy rate: 0.088), 4B (score 8–11, FP: 0.189–0.911, actual malignancy rate: 0.616), and 5 (score ≥ 12, FP: 0.798–0.999, actual malignancy rate: 0.951) (Fig. 3). The Cochran–Armitage trend test revealed that as the category increased, the FP also increased (*Z* = 22.239, *p* < 0.001). The AUC, accuracy, sensitivity, and specificity of the RSS in the training set were 0.883 (95% CI: 0.862–0.904), 0.826, 0.910, and 0.762, respectively. Supplementary Figs. 1–4 show STMs with RSS Categories 3–5. Furthermore, management recommendations were also proposed for each category according to the FP and clinical experience (Fig. 4). The malignancy proportion of Category 4A STMs with a score of 7 was significantly higher than that of Category 4A STMs with a score of ≤ 6 (*p* < 0.001). Observation was recommended for STMs in Categories 1, 2, 3, and 4A (score 3–6), and biopsy was recommended for STMs in Categories 4B–5. A thorough evaluation for a biopsy or observation was recommended when an STM reached a score of 7.

### External validation

For the validation set, the STMs malignancy rates with Categories 3, 4A, 4B, and 5 were 0% (0/5), 13.5% (7/52), 56.1% (23/41), and 95.5% (21/22), respectively. The AUC, accuracy, sensitivity, and specificity of the RSS in the validation set were 0.849 (95% CI: 0.778–0.920), 0.783, 0.863, and 0.725, respectively.

## Discussion

US is an effective imaging technique for STM management. Although previous studies have investigated the diagnostic value of US in STM diagnosis and developed diagnostic nomograms, they suffered from issues such as small sample sizes, ambiguous definitions of US features, complex calculations, and ignorance of clinical features. Moreover, previous studies have not yet conducted a US stratification system for STMs, which may confuse clinicians and limit clinical applications [15–17]. In order to effectively manage STMs, we built a practical RSS using the US and clinical features of 1027 STMs.

After standardizing the definitions of different US features, good inter-observer agreements were shown in the training and validation sets. Our findings revealed that age, duration, and history of malignancy in clinical features, and size, shape, margin, echogenicity, bone invasion, and vascularity in US features were indicative of malignancy, mostly aligning with previous studies [15, 16, 24–27]. In terms of clinical features, our study indicated that malignant STMs were independent of sex or location whereas previous studies found that malignant STMs were predominantly male and more likely to be located in the central parts of the trunks [15, 16, 27]. These discrepancies could be attributed to the different study sets in the respective studies. Additionally, patients with histories of malignancy were found to be more likely to suffer from malignant STMs in our study, possibly due to the considerable proportion of metastatic tumors in the training set. In terms of US features, tumor size, shape, margin, bone invasion, and vascularity were probably determined by the biological characteristics of malignant STMs, such as rapid growth, surrounding infiltration, and massive neovascularization [24, 27]. However, the association between echogenicity and malignancy remained controversial [16, 17, 24]. Our study indicated that predominantly hyperechogenicity or hypoechogenicity, rather than isoechogenicity, may indicate malignancy. Wu et al [28] reported that the mass echogenicity in the US could be related to the histopathologic compositions. Notably, hypoechogenicity was likely related to organized tumor cells [28], which is consistent with our study. Though hyperechogenicity was mainly related to benign compositions like adipocytes, cartilage, and osteoid tissues [28], atypical lipomatous tumors, classified as malignant STMs in our study, were histopathologically characterized as adipocytic variants and also appeared hyperechoic on the US [29]. Therefore, our study suggested hyperechogenicity remained a risk factor for malignant STMs.

As we know, our study was the first attempt to create an RSS for the diagnosis of STMs based on clinical and US risk factor weights. After assigning each risk factor a corresponding risk score calculated by standardized β coefficients, we found that as the total scores of STMs increased, both the FP and actual malignancy rates correspondingly increased. This trend was consistent with analogous studies in the thyroid reporting system [21, 30] and indicated the reasonableness of the RSS based on risk scores. In our study, STMs with different FP were classified into 6 categories given the low malignancy rates, the diverse pathological types, and the inherently overlapping US features of STMs [5]. Then, as the category level in the RSS increased, the corresponding FP and actual malignancy rates also increased. Thus, our RSS showed a generalizable diagnostic performance with an AUC value of 0.883 (95% CI: 0.862–0.904) in the training set and an AUC value of 0.849 (95% CI: 0.778–0.920) in the validation set. Management recommendations for different categories of STMs were made based on clinical practice and previous studies [31]. Similar to BI-RADS or TI-RADS, it was recommended in our RSS to perform biopsies on STMs with moderate (Category 4B) or high malignancy suspicion (Category 5) [22]. But for Category 4A in RSS, special attention was needed. Category 4A (score 3–7) was defined as low suspicion for malignancy in our RSS, but its FP range was wider than BI-RADS or TI-RADS. Specifically, in Category 4A, the malignancy rate of STMs with a score of 7 was higher than that of STMs with a score of 3–6, suggesting a need for separate analysis in the management of STMs with a score of 7. In all, clinical observation was recommended for STMs in Categories 1, 2, 3, and 4A (score 3–6), and biopsy was recommended for STMs in Categories 4B–5. For STMs in category 4A with a score of 7, comprehensive evaluation by clinicians was required. We believe that the RSS and corresponding management recommendations could have great value for the diagnosis and treatment of STMs.

Some limitations should be mentioned in our study. First, selection bias is inevitable due to the retrospective design with a limited dataset from three hospitals. The absence of pathological diagnoses for many STMs resulted in a relatively small sample size for benign STMs. Meanwhile, as a referral hospital, the proportion of malignant STMs in this study was far higher than the population-based incidence rate. Thus, a well-designed multicenter study is required to involve more STMs in a prospective setting. Second, the interpretation of images was inevitably subjective, despite the good consistency among observers. Involving more experts in the development and refinement of standard terms, together with applying artificial intelligence techniques to image acquisition and processing, could hopefully address this issue. Third, although our RSS showed good diagnostic performance, there was some overlap of FP between different categories. Fortunately, the potential value of multimodal US in the differential diagnosis of STMs has also been proved [10, 11, 26]. Accordingly, specifications for the acquisition and interpretation of images from the multimodal US should be developed, and a multimodal-US-based RSS could be constructed to improve diagnoses.

In summary, the practical RSS using both clinical and US characteristics may be a valuable tool in predicting STM malignancy, thereby promoting standardized management of STM by clinicians.

## Supplementary information

ELECTRONIC SUPPLEMENTARY MATERIAL

## Data Availability

The datasets used and/or analyzed during the current study are available from the corresponding author on reasonable request.

## Abbreviations

AUC: Area under the receiver operating characteristic curve
BI-RADS: Breast imaging reporting and data system
CDFI: Color doppler flow imaging
CI: Confidence interval
FP: Fitted probabilities
RSS: Risk stratification system
STMs: Soft tissue masses
TI-RADS: Thyroid imaging reporting and data system
US: Ultrasonography
WHO: World Health Organization
