# Supplementary material for: A practical risk stratification system based on ultrasonography and clinical characteristics for predicting the malignancy of soft tissue masses
Source: Insights Imaging. 2024 Sep 19;15:226. doi: 10.1186/s13244-024-01802-9 (PMC11424597; doi:10.1186/s13244-024-01802-9)
Supplement: Supplementary file 1 — ELECTRONIC SUPPLEMENTARY MATERIAL [file 13244_2024_1802_MOESM1_ESM.pdf]

A Practical Risk Stratification System Based on Ultrasonography and Clinical Characteristics for Predicting the Malignancy of Soft Tissue Masses

ELECTRONIC SUPPLEMENTARY MATERIAL

Supplementary Table 1. The inter-observer variability of US features in the training and validation sets.

Features	Training Set			Validation Set		
	Kappa	95% CI	Mass (n)	Kappa	95% CI	Mass (n)
**Layer**	0.851	0.562 - 1.000	50	0.959	0.865 - 1.000	50
**Shape**	0.960	0.872 - 1.000	50	0.891	0.703 - 1.000	50
**Margin**	0.840	0.676 - 0.960	50	0.769	0.516 - 0.947	50

**Echogenicity**	0.815	0.568 - 1.000	50	0.912	0.764 - 1.000	50
**Composition**	0.855	0.575 - 1.000	50	0.817	0.387 - 1.000	50
**Calcification**	0.855	0.588 - 1.000	50	0.912	0.631 -1.000	50
**Bone Invasion**	0.864	0.659 - 1.000	50	0.852	0.566 - 1.000	50
**Vascularity**	0.945	0.860 - 1.000	50	0.916	0.806 - 1.000	50

Note: CI, confidence interval; n, number; US, ultrasonography

Supplementary Figure 1. A STM classified as RSS category 3. (a) The mass is predominantly hyperechoic with a regular shape, a mean maximum diameter of 1.4 cm, a well-defined margin, and no bone invasion. **(b)** The mass is avascular. The patient is a 43-year-old male with a duration of over 12 months and no history of malignancy. The total score is 2. Postoperative pathology identifies it as a lipoma.

Note: RSS, risk stratification system; STM, soft tissue mass

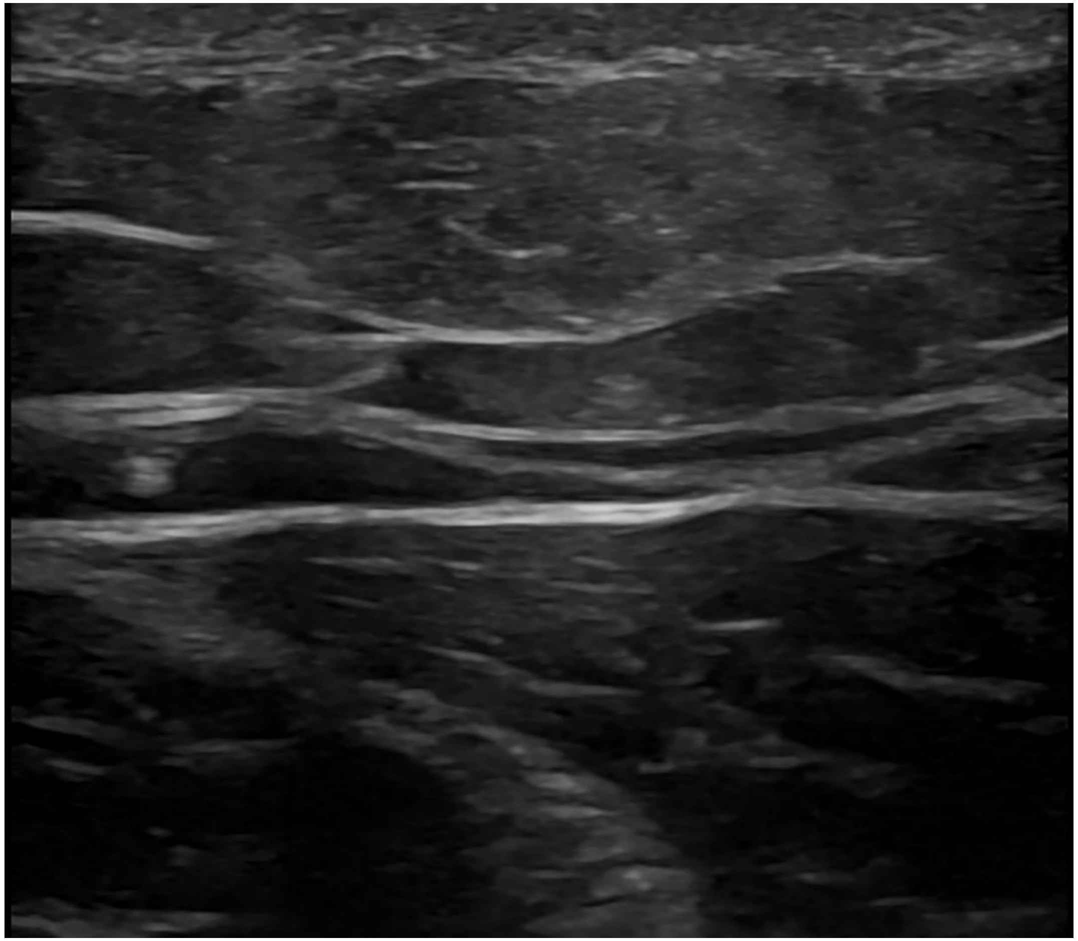

a.

b.

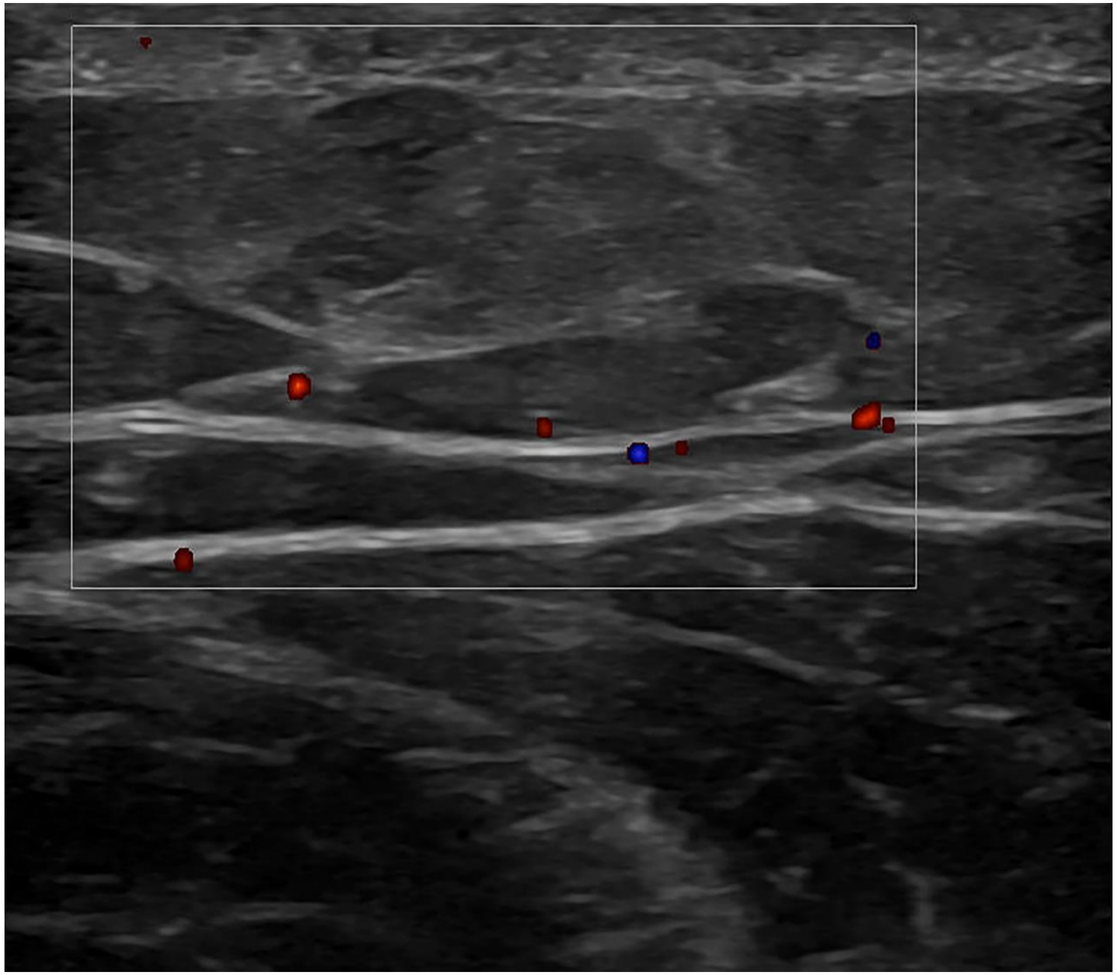

Insights Imaging (2024) Zhang YL, Wu MJ, Hu Y, et al.

Supplementary Figure 2. A STM classified as RSS category 4A. (a) The mass is predominantly hypoechoic with an irregular shape, a mean maximum diameter of 4.2 cm, a well-defined margin, and no bone invasion. **(b)** The mass has a grade I vascularity. The patient is a 38-year-old female with a duration of over 12 months and no history of malignancy. The total score is 5. Postoperative pathology identifies it as a schwannoma.

Note: RSS, risk stratification system; STM, soft tissue mass

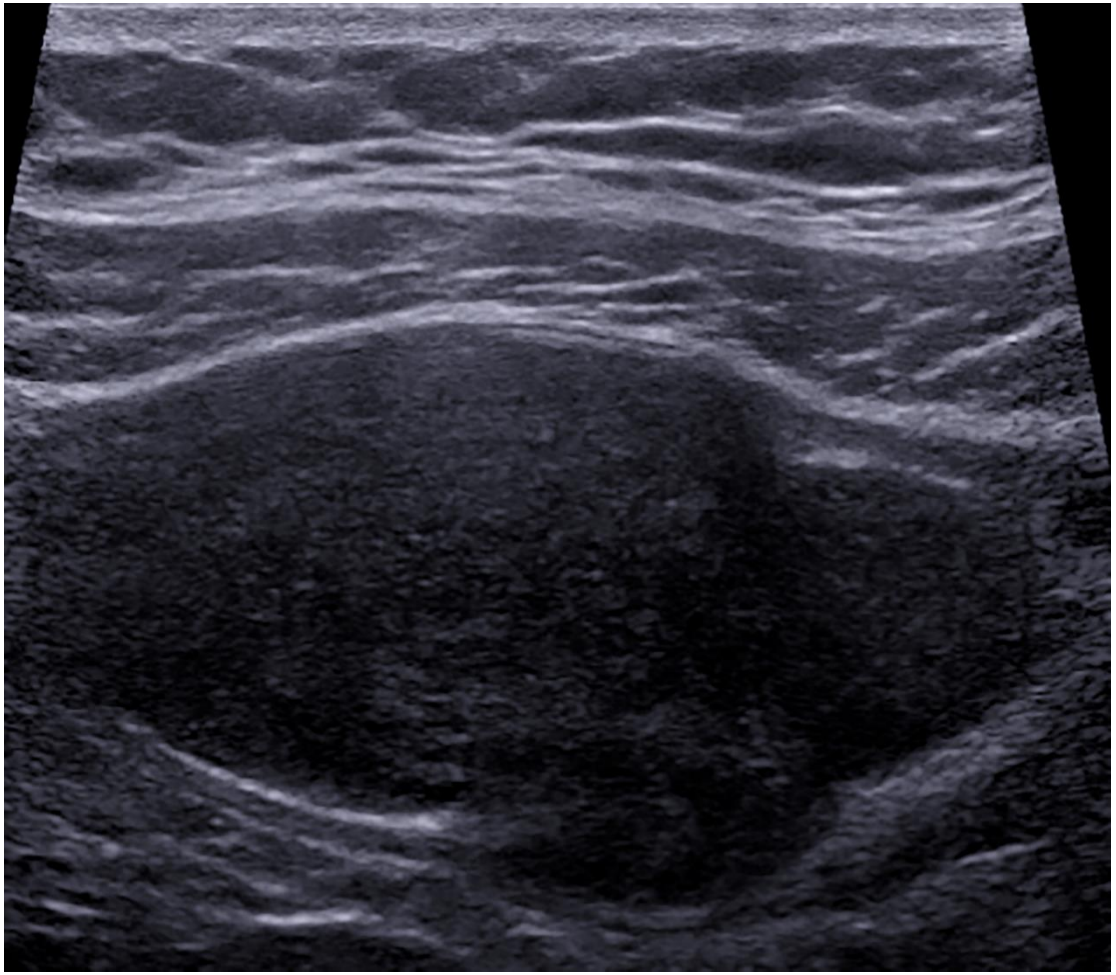

a.

b.

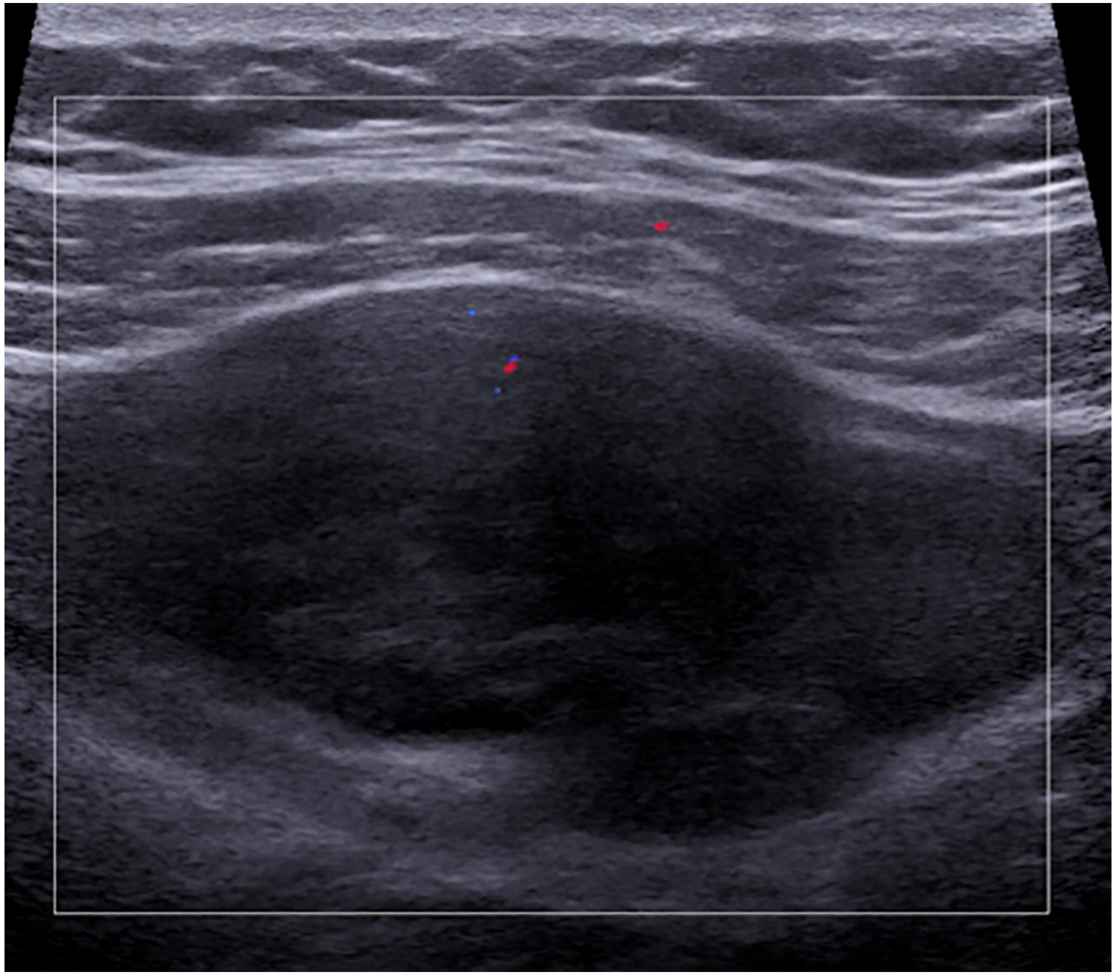

Supplementary Figure 3. A STM classified as RSS category 4B. (a) The mass is predominantly hypoechoic with a regular shape, a mean maximum diameter of 5.4 cm, a well-defined margin, and no bone invasion. **(b)** The mass has a grade III vascularity. The patient is a 43-year-old female with a duration of 20 days and no history of malignancy. The total score is 8. Postoperative pathology identifies it as a smooth muscle sarcoma.

Note: RSS, risk stratification system; STM, soft tissue mass

a.

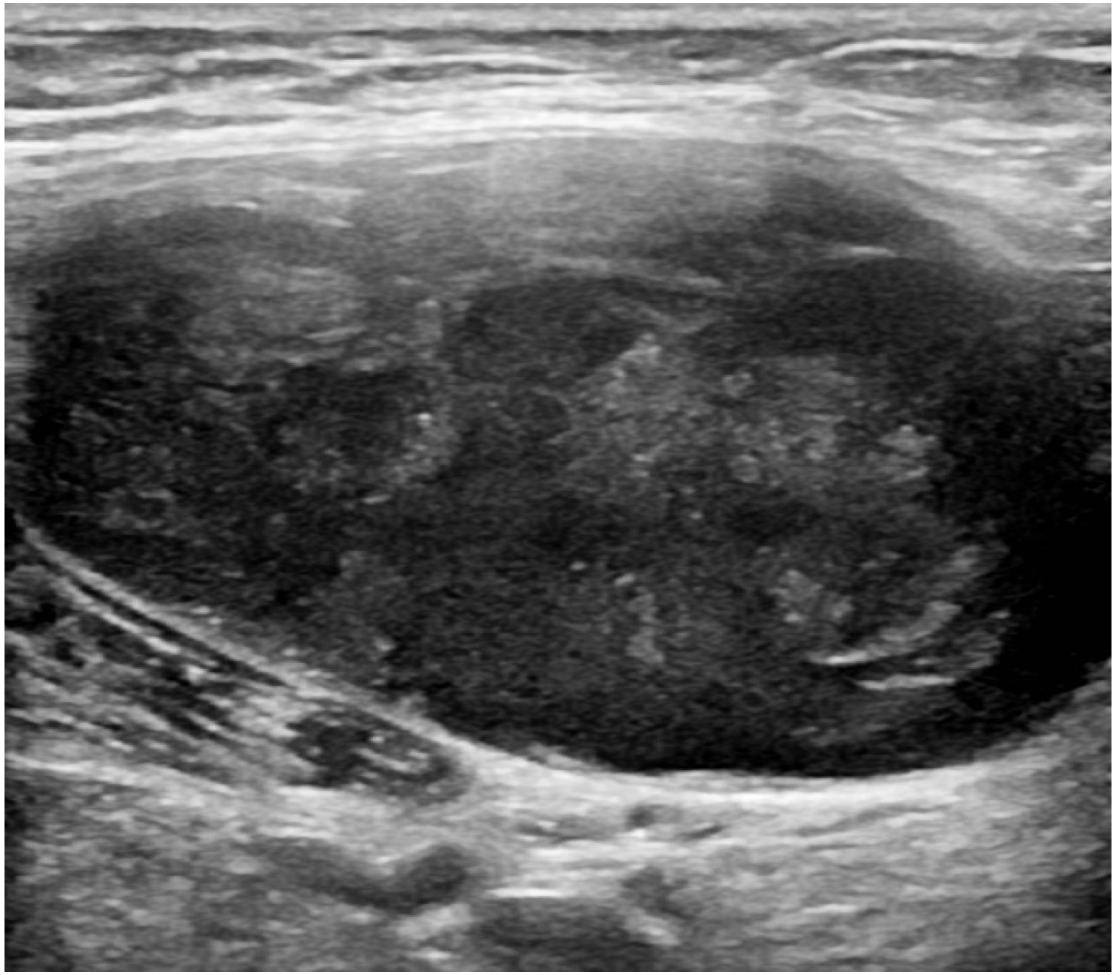

b.

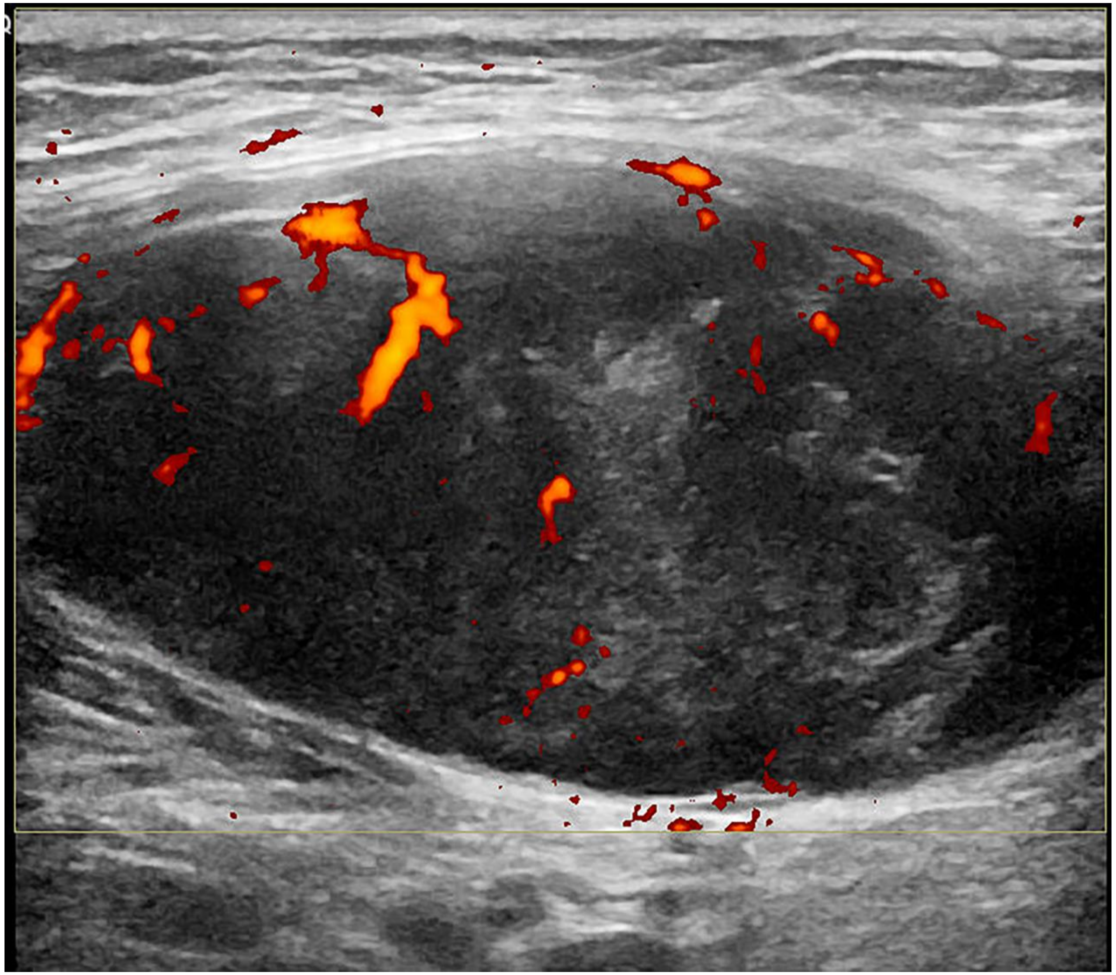

Insights Imaging (2024) Zhang YL, Wu MJ, Hu Y, et al.

Supplementary Figure 4. A STM classified as RSS category 5. (a) The mass is predominantly hypoechoic with a regular shape, a mean maximum diameter of 1.1 cm, an ill-defined margin, and no bone invasion. **(b)** The mass has a grade III vascularity. The patient is a 61-year-old female with a duration of 10 days and a history of malignancy. The total score is 12. Postoperative pathology identifies it as a metastatic carcinoma.

Note: RSS, risk stratification system; STM, soft tissue mass

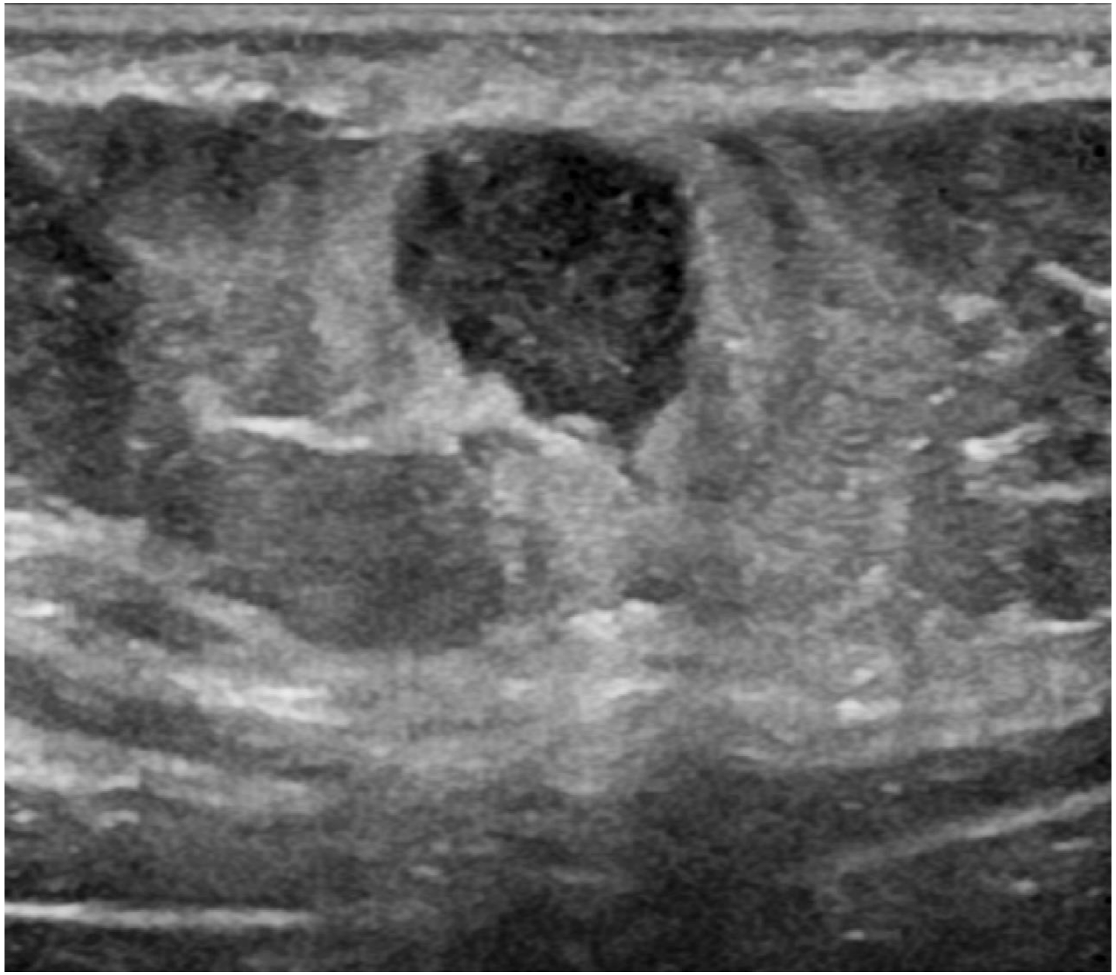

a.

b.

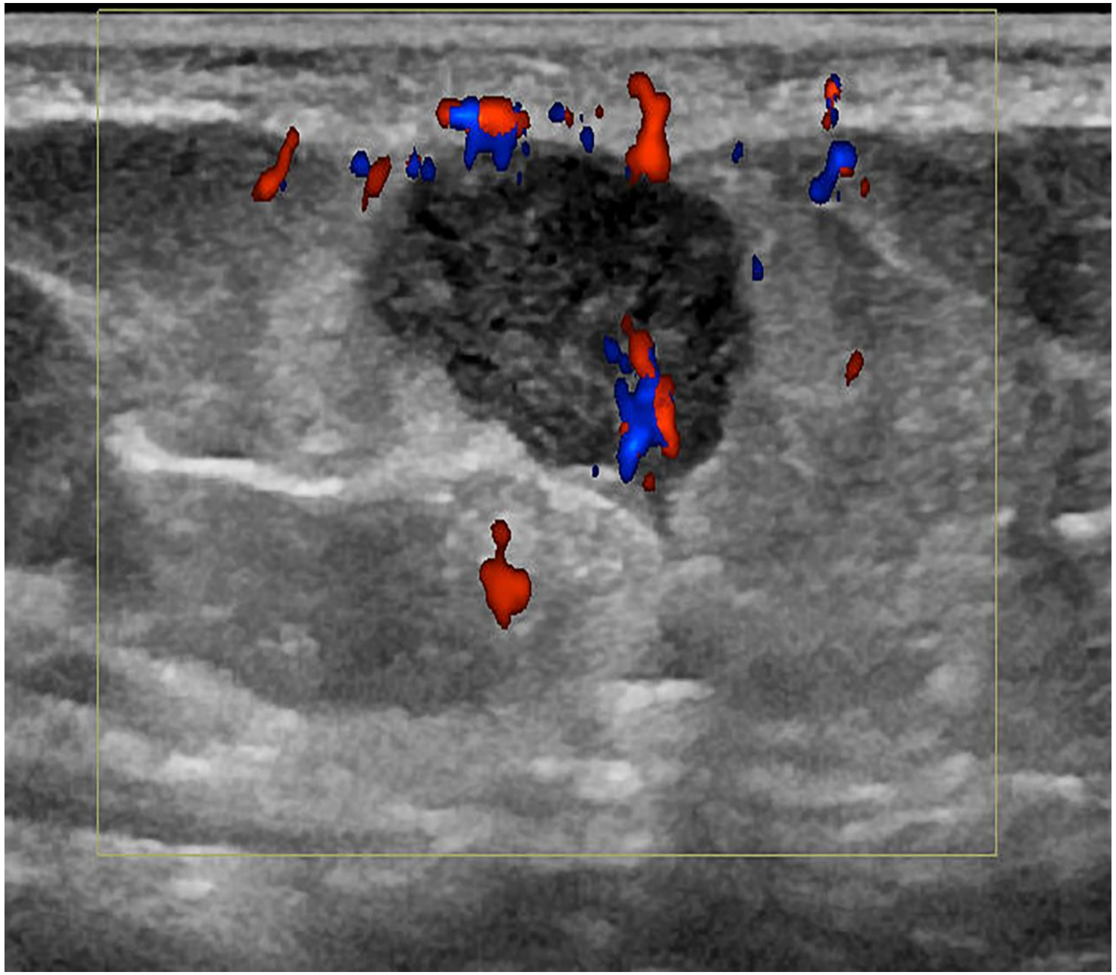

Insights Imaging (2024) Zhang YL, Wu MJ, Hu Y, et al.
